# Pivoting during the pandemic: A case study of the Senior Companion Program Plus

**DOI:** 10.1093/geroni/igab046.2964

**Published:** 2021-12-17

**Authors:** Noelle Fields, Ling Xu, Erin Roark, Sruthi Sundar, Ishan Williams, Joseph Gaugler

**Affiliations:** 1 University of Texas at Arlington, Arlington, Texas, United States; 2 The University of Texas-Arlington, Arlington, Texas, United States; 3 University of Virginia, Charlottesville, Virginia, United States; 4 University of Minnesota, Minneapolis, Minnesota, United States

## Abstract

Introduction: Growing research supports the use of older volunteers to provide respite and community-based assistance to persons with ADRD and their caregivers. This study explores the impact of COVID-19 on a face-to-face, peer-led psychoeducational intervention for African American ADRD family caregivers, the Senior Companion Program Plus (SCP-Plus), and its subsequent need to ‘pivot’ during the pandemic. Method: The SCP-Plus was a randomized control trial across three states that assessed program impact on ADRD family caregiver stress/burden, coping, and social support. In spring 2020, the SCP-Plus intervention was halted because of the potential risk to participants due to COVID-19 (n = 20 enrolled dyads). In an effort to maintain rapport and trust, critical to retention in research studies, team members began weekly (March-April) and then bi-weekly calls (May-December) for the purpose of providing a social check-in and to provide updates on the status of the intervention. Results: A total of 396 calls lasting approximately 10 minutes each were completed. Participants shared concerns around safety, access to food/supplies/masks/testing, feelings of stress and loss, concern for others, and the importance of technology as a means of social connection. Although the intervention aspect of the SCP-Plus ultimately ended due to COVID-19, information gleaned from these check-ins were used to pivot the study. The study moved forward by using a descriptive phenomenological approach to capture dyads’ lived experiences during COVID-19. Discussion: Overall, purposeful participant engagement through weekly/bi-weekly phone calls suggests that this is a promising strategy for participant retention as well as for pivoting research.

